# The Week's Work

**Published:** 1910-01-08

**Authors:** 


### THE WEEK'S WORK.

### CULPABLE LENIENCY.

MOST hospital superintendents of experience have found it necessary to avoid engaging ex-workhouse officials, because the principle on which a workhouse is too often run is what is known as the "good-enough" policy. That is to say, however badly done work may be, there are frequently members of the Board of Guardians, and sometimes a majority, who vote against the punishment of the offenders, because, apparently, they hold that anything is good enough for paupers. What, for instance, is the explanation of two decisions arrived at by the West Ham Guardians, as reported in the *Essex Times* of December 29? In the first case, the butter and margarine supplied to the inmates by the storekeeper, to the extent as stated by one member of 68 lb., was found to be "vile and uneatable." The chairman stated it should be known that the officer responsible had been in the habit of accepting stores in a form other than that laid down by the guardians. The House Committee recommended that this officer's resignation should be called for. The majority of the board, however, appeared to attach little importance to this cruel treatment of the defenceless inmates of the workhouse, and sent the report back to the committee. We hope the members of the committee will stand firm to the sound principle which they laid down, as we understand them, which is that an officer who accepts stores contrary to regulations and accepts supplies from contractors of uneatable food for the inmates of a workhouse should be called upon to resign.

### WORKHOUSE CHILDREN.

WE recently called attention to the unhappy lot of many children in Poor Law infirmaries, but the lot of the poor children at West Ham seems also to be cruel too. The chairman of the Infirmary Visiting Committee having reported that the medical superintendent recommended that a probationer nurse should forthwith relinquish her duties for contravening an order of the board by striking a child, and further that the medical superintendent had found it necessary to put up an additional notice on account of the threats used by the nurses to the children in this infirmary, the majority of the board refused to ratify the recommendation removing the probationer, and adjourned the discussion. Clearly, unless the board at its next meeting supports its medical superintendent he must decline to be further responsible for the treatment of the children in this Poor Law infirmary. When a board of guardians is responsible for decisions of this kind in matters of urgency and importance to the whole efficiency of the establishments under their control, it is time for the Local Government Board to intervene, as it did at Poplar. The action of the majority of the West Ham Guardians is one more striking proof of the wisdom of the recommendation of the Poor Law Commission to abolish boards of guardians altogether.

### THE SATURDAY FUND.

SIR SAVILE CROSSLEY presided at the quarterly meeting of the board of delegates of the Hospital Saturday Fund, when it was reported that the receipts from the industrial establishments from January 12 last reached nearly £22,500 —or about the same amount as at this period last year. The accounts for 1909 will close on Monday next, the 10th. The sum of £27,000 has been fixed as a basis for the forthcoming awards. The report of the council, which was adopted, showed that during this quarter 13,895 letters of recommendation to the various medical charities had been issued. These included 301 convalescent home letters. The dental sub-committee in this period considered 684 applications for dental treatment, and made grants amounting to £302 7s. 9d. to 585 applicants. The remaining cases, 85, were adjudged to pay the full cost. In the surgical appliance department 2,375 attendances were made by patients who contributed towards their appliances £296 18s. 10d. The waiting lists for admission to the endowed beds at the consumption hospitals were not so long as last year, owing to the increased accommodation provided by the Fund. The Council regretted that there was still a waiting period, especially for women, at hospitals where the applicants were relying upon free letters.

### UNIFORM SYSTEM OF HOSPITAL ACCOUNTS.

WITH a view to lightening as far as possible the labours of hospital officials charged with the duty of supplying manuscript information in connection with applications for grants, King Edward's Hospital Fund, the Metropolitan Hospital Sunday Fund, and the Hospital Saturday Fund have decided that their questions as to "statistics," "income and expenditure," and "salaries and wages" at hospitals, convalescent homes, and similar institutions shall be uniform in wording, so that the replies drafted for one fund can be copied, when the time comes, on to the forms issued by the other two. This will apply only to these particular questions, and not to inquiries upon other subjects. Nor will it apply to nursing institutions or dispensaries. An index of classification for 1910, with supplementary notes, has been issued.

### THE NEW HOUSE GOVERNOR AT STOKE.

THE Committee of the North Staffordshire Infirmary did not re-advertise the vacancy caused by the death of Dr. Roscoe. Instead, they invited the attendance of certain former applicants, who included Lieut.-Col. Mills and Major Williams (both of the Indian Medical Service), Fleet-Surgeon Nance, R.N., Mr. J. S. Neil (House Governor of the Wolverhampton and Staffordshire General Hospital), Dr. T. B. Rhodes (Superintendent of the Baguley Fever Hospital), and Mr. William Stevenson (Secretary of the Devonshire Hospital, Buxton). Two of the candidates, Messrs. Neil and Stevenson, have had experience in the raising of money and of the financial and business work connected with a voluntary hospital. We must once again emphasise the importance of selecting men of special experience and training for every responsible post in a voluntary hospital. In the result the Committee, by the votes of the majority, elected Mr. T. Basil Rhodes, M.B., B.S., Durham, Medical Superintendent of the Baguley Fever Hospital. In congratulating Mr. Rhodes, we hope he may find the work agreeable and well within his knowledge, experience and compass.

### OUT-PATIENTS' CARDS.

The first of the recent inquests in connection with the deaths of certain out-patients of the Battersea Anti-Vivisection Hospital drew attention to the question of providing printed cards of instructions for the benefit of patients. With the other questions raised at these inquests we deal elsewhere, but this point is one which demands a little more detailed consideration. In all properly regulated institutions such cards are provided. They give, in simple and concise language, the gist of the instructions verbally given by the dresser or house surgeon to the patient, and they are therefore a safeguard alike to the hospital and to the patient. Should any question afterwards arise—as may happen in the best regulated hospitals—the printed card is usually available for reference, and as an added safeguard it should have the name and address of the patient written on it, and should be initialled by the dresser or nurse. There can be no excuse for neglecting to provide such cards, as appears to be the case at the Battersea Anti-vivisection Hospital. The expense of printing them is relatively small, and their usefulness is almost too well known to need comment. The facts brought out at the recent inquest show clearly that the institution in question can scarcely be considered up to date or that it is managed in the most approved fashion.

### A RECORD OF OPERATIONS

At a recent board meeting of a provincial hospital, considerable discussion arose upon the question of allowing a detailed record of all operations performed in the institution to be laid before the board, and to allow a statistical abstract of such record to be published in the annual report. It appears that a lay member of the board thought that it was desirable that the members should have some such analysis before them, in order to know what was going on, and the relative amount of work each member of the staff was doing. This suggestion was hotly opposed by other lay members and by the medical representatives on the board, and the discussion, we are sorry to say, was carried beyond the walls of the committee room and intruded into the columns of the local press. The question at issue appears to be a very important one, but it is one that can only be thoroughly debated by those who are acquainted with hospital matters, and who can correctly weigh and appreciate the arguments on both sides. In an institutional paper such discussion is, however, quite allowable, and even desirable, and we will be glad to get our readers' opinions on the subject.

### THE CASE FOR THE RETURNS.

The main argument advanced in favour of the practice of submitting such an analysis of operations to the committee of management is that it affords a ready means of gauging the extent of the surgical work of the hospital. The surgical staff has no other opportunity of putting before the board concrete evidence of its activity, and members of the committee have therefore no means of correctly estimating and appreciating the work of each individual member of the staff unless such a return is made. The committee has the right to demand such analysis: it has the moral right to judge for itself the efficiency of each individual member of the staff—nay, more, it has a duty to perform in demanding returns which can enable it so to judge. The returns of length of stay in the surgical wards afford no true criterion: one bad case may invalidate the correctness of any conclusion drawn from such figures. Again, the mortality figures give no helpful evidence either way. A surgeon who does his work badly and inefficiently may yet prove a lower mortality figure than his colleague can show, even though the latter is more expert, more aseptic, and more efficient in every way. Conclusions drawn from the length of stay of each patient in the wards are, as already said, manifestly open to objections. One surgeon may hurry his cases out and readmit them a week or two later: his results may be bad, his percentage of septic cases may exceed 10 per cent., and yet his time average may be well under twenty days. His colleague, on the other hand, may tackle far worse cases, and may conscientiously keep two or more such in his wards until they are really improved, with the result that his average rises to thirty days or more. Where no operation return is made, the committee of management has no means of judging between these statistics: where such a return is available the fallacies in the one set of conclusions become manifest as soon as the figures are compared with the operation returns. For that reason the operation returns in most German hospitals are full and clear. Not only the nature of the operation, but its result as well is given impartially, as the following shows: "No. 3906. Heindrich Hincke: Double inguinal hernia. Bassini's operation: healing by primary intention left side: suppuration (superficial), sloughing round stitches right side. Discharged cured. Operator, Dr. ——. Note.—In consequence of the slight infection of this patient's wound his length of stay in the ward was prolonged nine days beyond the usual average. The cause of the suppuration was found to be ——." Such a return is unusually full, and entails a considerable amount of extra clerical work, but it is of decided value in every case. At most of the London hospitals an "operation list" is furnished at every board or committee meeting. In such operation list the number and nature of operations are specified, and a note is made if any postponement in the case of patients prepared for operation has taken place. Where the percentage of such postponements exceeds the normal, the surgeon responsible for them is promptly written to and asked to explain. In that way the committee has a certain guide before it, and is able to estimate pretty accurately the proportionate amount of work done. All this makes for efficiency, and enhances the thoroughness in attention to detail which should be characteristic of every modern hospital worthy of being considered up to date.

### THE CASE AGAINST.

On the other hand, those who oppose the compilation of such returns do so on grounds that appear to be largely sentimental. They allege, in the first place, that a committee of management should have supreme confidence in its staff, that to demand such a return is casting a slur upon the members of the surgical staff by doubting their efficiency, and that the real value of such a compilation is problematical. They argue that no lay committee can be expected to judge of the operative skill or professional efficiency of a surgeon, and that conclusions from results are wholly inaccurate in the case of operations. Finally, they consider that the publication of such a return, even before the board of management alone, is calculated to damage the reputation of the institution, since in such returns there must at times figure very bad and discouraging cases. All these arguments are in a measure true, but none of them seem to us to weigh very strongly against those in favour of compiling such returns. There is no ethical reason, contrary to what has been alleged, why such returns should not be laid before the committee, since that is the method already followed in the best London and provincial hospitals. It is true that by appointing a surgeon to its staff a hospital board shows its confidence in such a man, but that confidence is not absolute, and cannot, if the institution is to be efficient, be irrevocable. In the best interests both of the public and of the profession, to say nothing whatever of the hospital itself, it is desirable that the hospital surgeons' work should be able to bear the fullest light of day. Where such returns, therefore, are opposed by the staff, it is presumptive evidence that there is something wrong. No member of a lay committee—presuming that the board of management is entirely composed of lay members, which is very rarely the case—can judge of the nature and results of an operation; but every layman who has any responsibility to share in the management of a hospital ought to be able to judge from the gross results whether or not the surgical staff is as efficient as it ought to be. Unless he is able to do this he should not be allowed to meddle in the affairs of the hospital at all. We have not the slightest doubt that a compilation of operations such as is made by every reputable modern hospital for the consideration of its committee of management is of the utmost service in affording members of the committee an opportunity of gauging the general efficiency and thoroughness of the work of the institution.

### THE DRUG MARKET.

Although business in the drug and chemical markets is not yet "in full swing," more interest is being taken in several articles than is usual during the first week of a new year, and a fairly healthy tone pervades the markets. The general impression prevails that business will improve very considerably, and there are not wanting signs that in the near future there will be an end of the inactivity which has characterised the drug market for many months past. An increased demand is usually accompanied by a greater number of price fluctuations, and it is therefore advisable to be on the look-out for indications of alterations in value. At the moment there seems a possibility that quinine, the market in which has been stagnant so long, will advance in price, as a strong effort is being made by the Java planters of cinchona bark to limit their output; the advance may not be immediate, but it is highly improbable that the price of quinine during the present year will be lower than it is now. Carbolic acid is another article which may be regarded as having reached the lowest price. Cod-liver oil is tending dearer, as also is menthol. Buchu leaves are fetching very high prices, having advanced 25 per cent. within a week or so.

